# The MAPKKK gene family in cassava: Genome-wide identification and expression analysis against drought stress

**DOI:** 10.1038/s41598-017-13988-8

**Published:** 2017-11-02

**Authors:** Jianqiu Ye, Hai Yang, Haitao Shi, Yunxie Wei, Weiwei Tie, Zehong Ding, Yan Yan, Ying Luo, Zhiqiang Xia, Wenquan Wang, Ming Peng, Kaimian Li, He Zhang, Wei Hu

**Affiliations:** 1 Tropical Crops Genetic Resources Institute, Chinese Academy of Tropic Agricultural Sciences, Danzhou, Hainan China; 2 Key Laboratory of Biology and Genetic Resources of Tropical Crops, Institute of Tropical Bioscience and Biotechnology, Chinese Academy of Tropical Agricultural Sciences, Haikou, Hainan China; 3 Environment and Plant Protection Institute, Chinese Academy of Tropical Agricultural Sciences, Haikou, Hainan China; 4 Hainan Key Laboratory for Sustainable Utilization of Tropical Bioresources, College of Agriculture, Hainan University, Haikou, Hainan China; 5 College of Life Science and Technology, Huazhong University of Science & Technology (HUST), Wuhan, Hubei China

## Abstract

Mitogen-activated protein kinase kinase kinases (MAPKKKs), an important unit of MAPK cascade, play crucial roles in plant development and response to various stresses. However, little is known concerning the MAPKKK family in the important subtropical and tropical crop cassava. In this study, 62 MAPKKK genes were identified in the cassava genome, and were classified into 3 subfamilies based on phylogenetic analysis. Most of MAPKKKs in the same subfamily shared similar gene structures and conserved motifs. The comprehensive transcriptome analysis showed that *MAPKKK* genes participated in tissue development and response to drought stress. Comparative expression profiles revealed that many *MAPKKK* genes were activated in cultivated varieties SC124 and Arg7 and the function of *MeMAPKKK*s in drought resistance may be different between SC124/Arg7 and W14. Expression analyses of the 7 selected *MeMAPKKK* genes showed that most of them were significantly upregulated by osmotic, salt and ABA treatments, whereas slightly induced by H_2_O_2_ and cold stresses. Taken together, this study identified candidate *MeMAPKKK* genes for genetic improvement of abiotic stress resistance and provided new insights into MAPKKK -mediated cassava resistance to drought stress.

## Introduction

To survival, plants have elaborated signaling network involved in sensing and transmitting signals. Among the stress-activated molecular pathways, mitogen-activated protein kinases (MAPKs) signaling cascade plays central roles in multiple biological processes^1,2^. The basic MAPK cascade is consist of three classes of protein kinases, including MAPK kinase kinase (MAPKKK), MAPK kinase (MAPKK) and MAPK, which are linked to upstream and downstream regulators by phosphorylation^3^.

In recent years, studies have suggested that MAPK cascade is involved in various biological processes in plants, including cell differentiation and growth^4,5^, plant ripening, hormone signaling^6–8^, plant immunity^1,9^, and respondent to the multiplicity of biotic and abiotic stress^10–13^. Arabidopsis MEKK1-MKK4/5-MPK3/6 cascade participates in innate immunity, which is the first identified MAPK signal pathway in plants^1,14^. Subsequently, a series of MAPK signal pathways are defined. Tobacco NPK1-MEK1-Ntf6 plays an important role in resistance to tobacco mosaic virus^15^. The MEKK1-MKK2-MPK4 cascade takes part in cold stress response^16^. Arabidopsis ANP3-MKK6-MPK4 and YDA-MKK4/5-MPK3/6 are involved in the regulation of cytokinesis and stomatal development, respectively^17,18^. Besides, some genes related to MAPK cascade pathway are identified to play a vital role in environmental stress responses. For example, *MAP3Kd4* plays an important role in increasing salt stress resistance and ABA response by over-expression analysis^11^. Mutation of *raf43–1* results in increased sensitivity to mannitol, NaCl and H_2_O_2_^19^. Another Arabidopsis Raf-like MAPKKK gene, *HT1*, positively regulates response to CO_2_^20^. A cotton Raf-like MAPKKK gene, *GhRaf19*, positively regulates resistance to cold stress, but negatively regulates resistance to drought and salt stresses using virus-induced gene silencing^21^. Overexpression of tobacco *NPK1*, belonging to the MAPKKK family, enhances maize resistance to drought^22^. Together, the above studies support that MAPK cascade pathway is a crucial regulator of plant development and stress response.

To date, many *MAPKKK* genes have been identified in several plant species based on genome sequencing, including *Arabidopsis* (80 members)^23^, *Oryza sativa* (75 members)^24^, *Zea mays* (71 members)^25^, *Lycopersicon esculentum* (89 members)^26^, *Cucumis sativus* (59 members)^27^, *Glycine max* (150 members)^28^, *Medicago truncatula* (73 members)^29^, *Musa acuminate* (77 members)^30^, and *Vitis vinifera* (45 members)^31^. Currently, there is little information concerning the MAPKKK family in cassava that is an important crop across the tropical and sub-tropical world^32–36^. Although cassava has the characteristic of drought resistance, the underlying mechanism is elusive because of the limitation of cassava cultivation zones. Thus, investigation of the gene families related to MAPK cascade is necessary to elucidate the biological processes of cassava development and stress responses.

In this study, we identified 62 *MeMAPKKK*s from the cassava genome, and further investigated their phylogenetic relationship, protein motifs, gene structure, expression patterns in diverse tissues, and the responses to drought stress in three cassava accessions. This comprehensive study will increase our understanding of MAPKKK associated with the developmental process and abiotic stress responses, and build a solid foundation for future studies aimed at MAPK signaling cascade-mediated genetic improvement of cassava.

## Results

### Identification and phylogenetic analysis of cassava MAPKKK family

By BLAST and Hidden Markov Model searches, a total of 62 *MeMAPKKKs* were obtained from the complete cassava genome with Arabidopsis and rice MAPKKK sequences as queries. The 62 predicted MAPKKK proteins ranged from 284 (MeMAPKKK17) to 1021 (MeMAPKKK33) amino acids (aa) in length with an average of 612.2 aa, the molecular weight (MW) varied from 32.5 kDa (MeMAPKKK17) to 111.8 kDa (MeMAPKKK33), and the theoretical isoelectric point (pI) ranged from 4.74 (MeMAPKKK61) to 9.5 (MeMAPKKK5) with 49 numbers pI <7 and the others pI <7 (Table S1).

**Figure 1:**
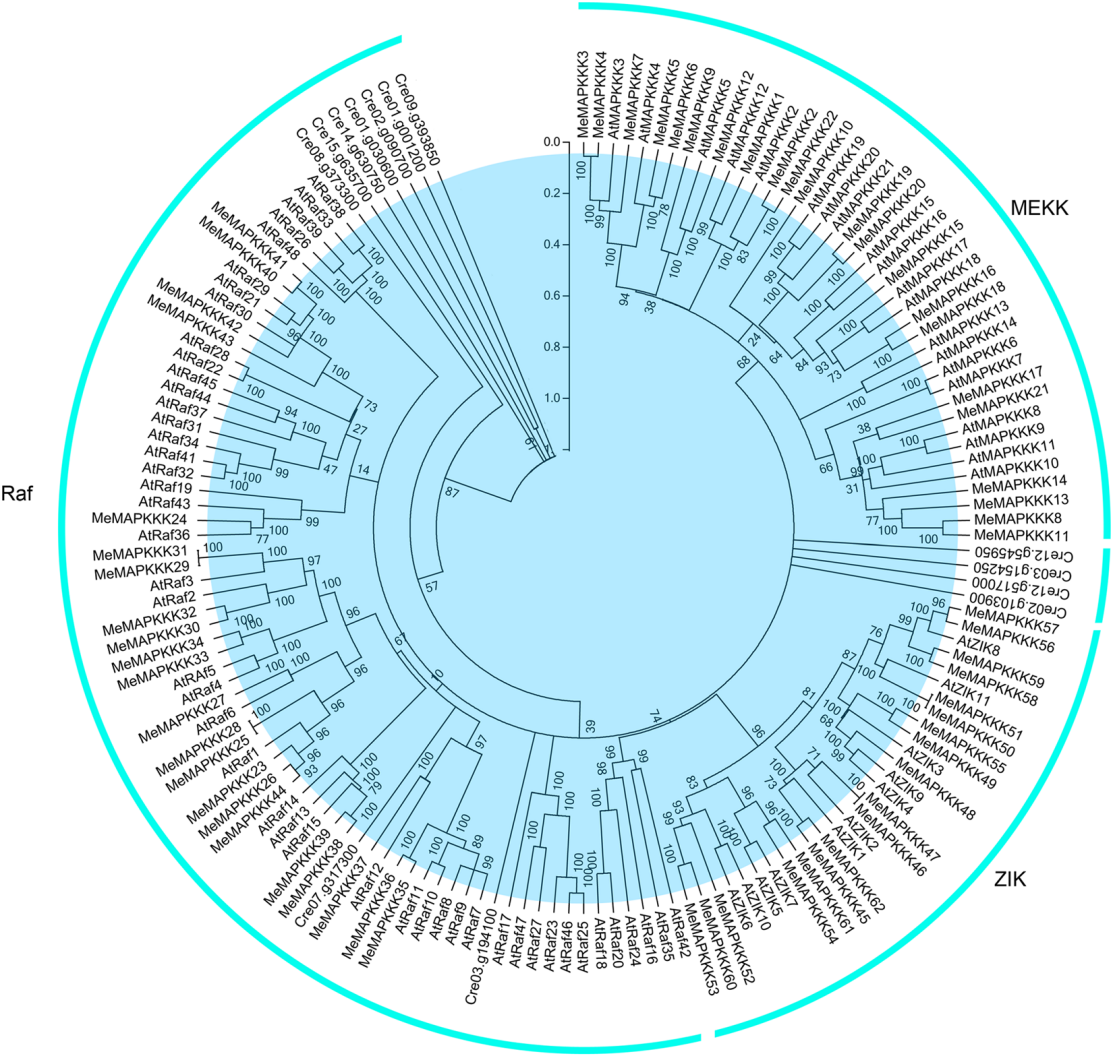
Phylogenetic analysis of MAPKKKs from cassava, Arabidopsis, and *Chlamydomonas reinhardtii* using the complete protein sequences. The Neighbor-joining (NJ) tree was reconstructed using Clustal X 2.0 and MEGA 5.0 softwares with the pair-wise deletion option. 1000 bootstrap replicates were used to assess tree reliability.

To investigate the evolutionary relationships of MAPKKK proteins between cassava and Arabidopsis/*Chlamydomonas reinhardtii*, a phylogenetic tree was constructed after alignment of amino acid sequences of all 155 MAPKKKs (62 in cassava, 80 in Arabidopsis, and 13 in *Chlamydomonas reinhardtii*) using Clustal X 2.0 and MEGA 5.0 softwares. As indicated in Fig. 1, 62 MeMAPKKK proteins were grouped into three clusters, namely Raf, ZIK and MEKK subfamily, in accordance with the categories of MeMAPKKKs in Arabidopsis. There were 22 MeMAPKKKs, 48 AtRAFs and 9 MAPKKKs from *Chlamydomonas reinhardtii* in the Raf subfamily, 18 MeMAPKKKs and 11 AtZIKs in the ZIK subfamily, and 22 MeMAPKKKs and 21 AtMAPKKKs in the MEKK subfamily. The additional 4 MAPKKKs in *Chlamydomonas reinhardtii* were not clustered, suggesting their independent evolution. Phylogenetic analysis also showed that there were some closely related orthologous MAPKKKs between cassava and Arabidopsis, suggesting that an ancestral set of MAPKKK genes existed before the divergence of cassava and Arabidopsis.

### Conserved motifs and gene structure of *MeMAPKKKs*

**Figure 2:**
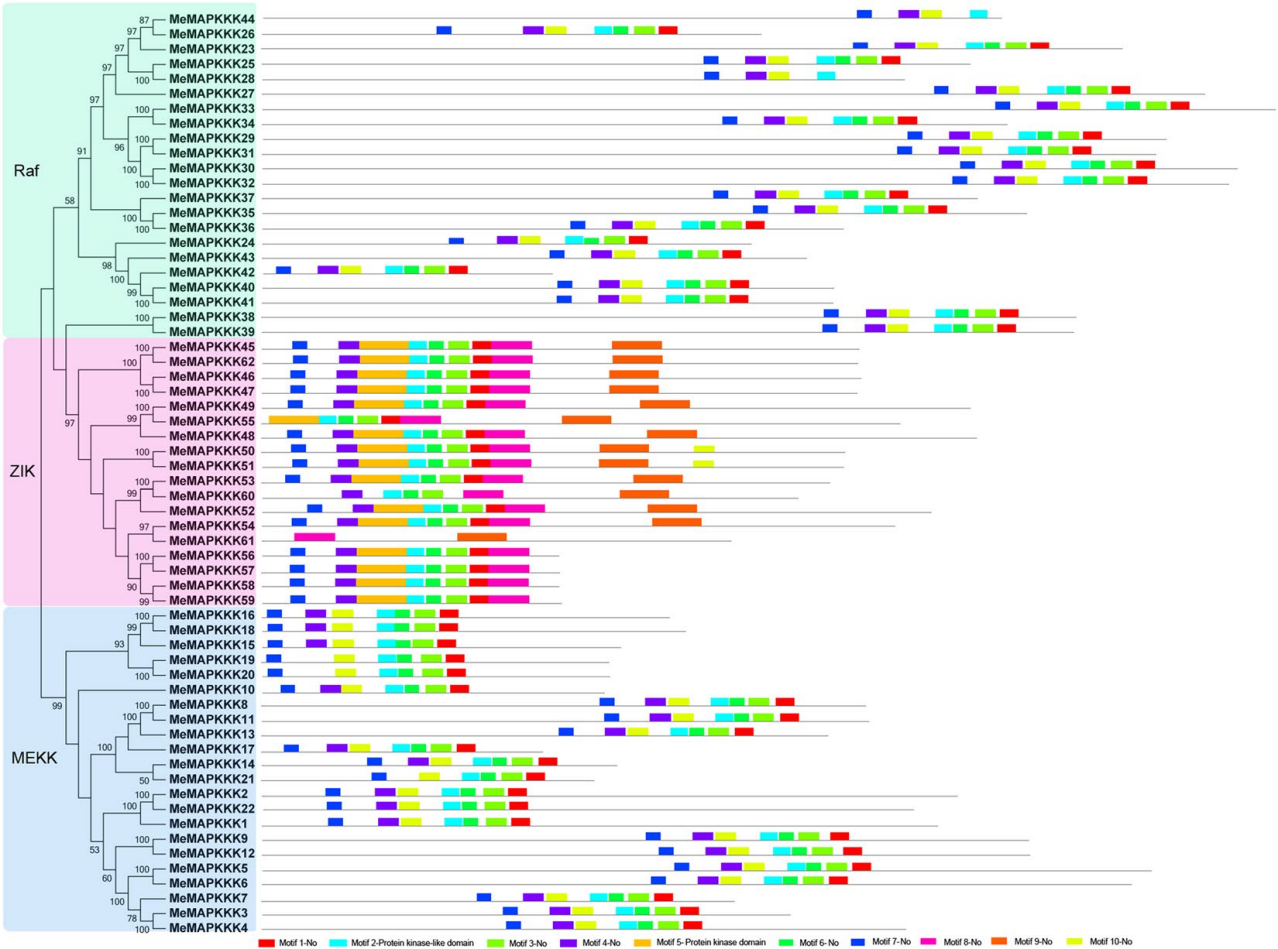
The conserved motifs of cassava MAPKKKs according to phylogenetic relationship. All motifs were identified by MEME database with the complete amino acid sequences of cassava MAPKKKs.

To further understand the structural features of MeMAPKKKs, 10 conserved motifs were identified using MEME software and further annotated by InterPro Scan 5 (Fig. 2). Motif 2 and Motif 5 were marked as protein kinase domain, while the remaining 8 motifs had no annotation. All the identified MeMAPKKKs had motif 2, and all the ZIK subfamily members specially showed motif 5, expect for MeMAPKKK61. Additionally, all the MeMAPKKKs, except for MeMAPKKK19, -20, -21, -28, -44, -60, and -61, contained motifs 1–4. Motif 8 only presented in ZIK subfamily, while motif 10 uniquely displayed in Raf and MEKK subfamily. These results suggest that each subfamily of MAPKKKs shares the similar protein structure and domain composition, suggesting that protein architecture is dramatically conserved within a specific subfamily of MAPKKKs.

**Figure 3:**
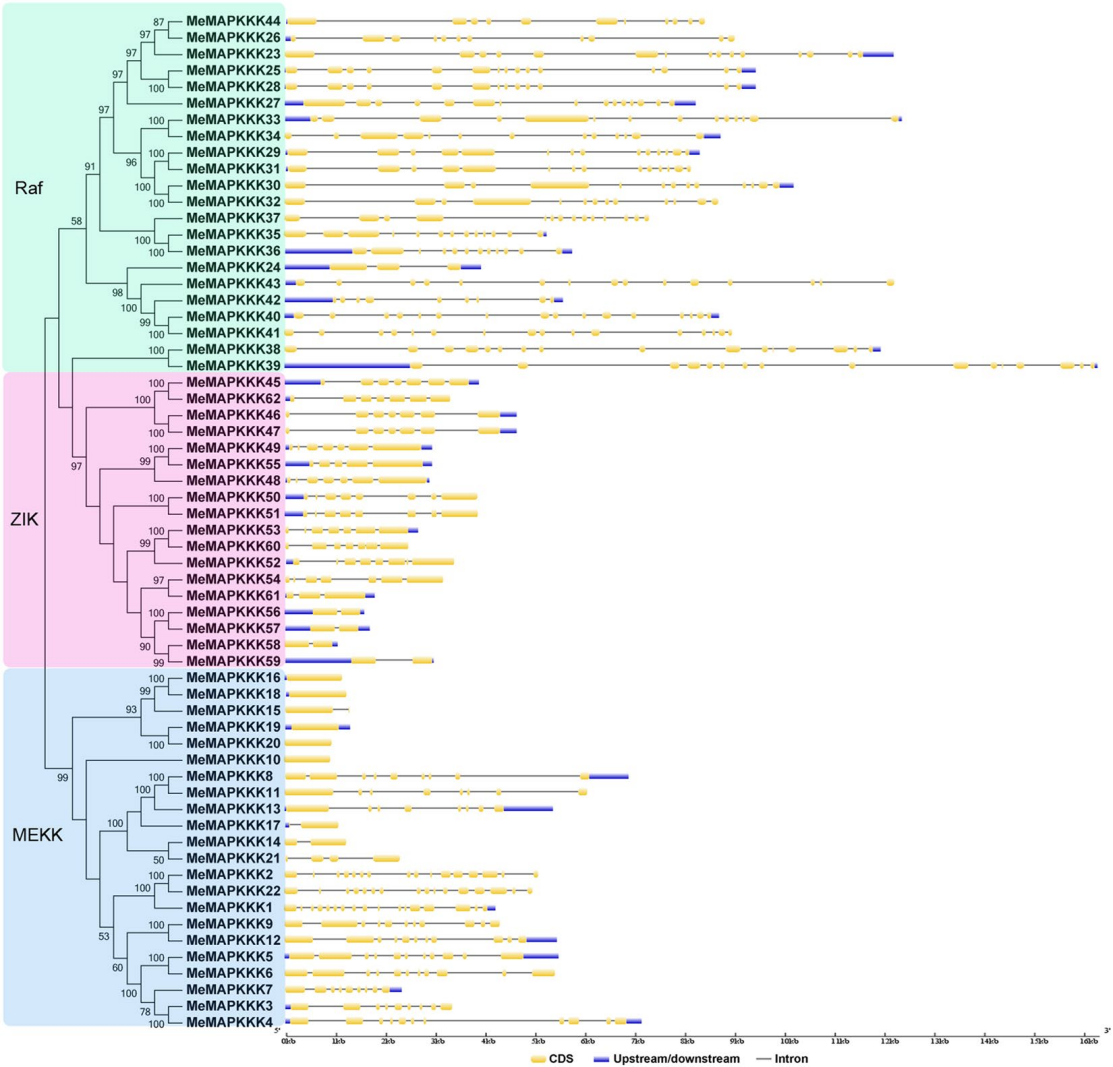
Gene structure analyses of cassava *MAPKKKs* according to phylogenetic relationship. Exon-intron structure analyses were performed by GSDS database. The blue boxes, yellow boxes, and the black lines indicate upstream/downstream, exons, and introns, respectively.

To reveal the structural features of *MeMAPKKKs*, intron-exon organizations were analyzed by Gene Structure Display Server (GSDS) v2.0. The number of intron in *MeMAPKKKs* varied from 0 (*MeMAPKKK16*, *-18*, *-15*, *-19*, *-20*, and *-10*) to 16 (*MeMAPKKK1*) (Fig. 3). Most of *Raf* subfamily genes contained more than 10 introns, while the intron in *ZIK* group genes varied from 1 to 7. Generally, *MeMAPKKKs* in the same cluster of the phylogenetic tree show similar exon-intron structures, indicating the link between evolutionary relationship and gene structure.

### Expression profiles of *MeMAPKKKs* in different tissues of two cassava genotypes

**Figure 4:**
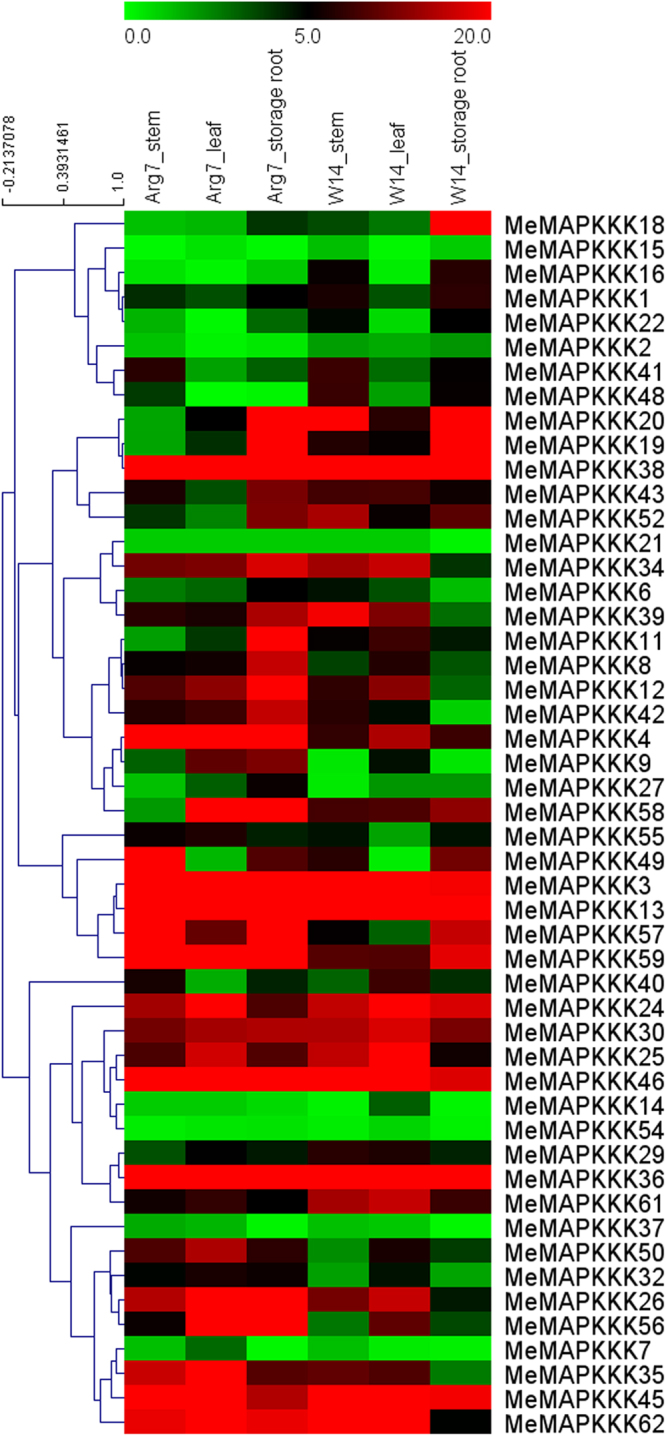
Expression profiles of cassava *MAPKKKs* in stems, leaves, and storage roots of Arg7 and W14. The heat map was constructed according to the FPKM value of cassava MAPKKKs. FPKM value is shown in color as the scale.

To detect the possible role of *MeMAPKKKs* in cassava growth and development, the stems, leaves and storage roots of wild subspecies (W14) and cultivated variety (Arg7) were collected for RNA-seq analyses. 80% (50/62) of *MeMAPKKK* genes were captured from the RNA-seq data (Fig. 4; Table S2). Generally, 13 out of 50 *MeMAPKKK* genes had high expression levels (value >5) in the tested tissues of the two genotypes, such as *MeMAPKKK3*, *-4*, *-12*, *-13*, *-24*, *-25*, *-30*, *-36*, *-38*, *-43*, *-45*, *-46* and *-59*. In contrast, 8 *MeMAPKKK* genes (*MeMAPKKK2*, *-7*, *-14*, *-15*, *-21*, *-22*, *-37*, and *-54*) showed low transcript abundance (value <5) in the tissues of the two accessions.

For Arg7 varieties, 26 of 50 *MeMAPKKKs* showed higher transcripts in storage roots, relative to that in stems or leaves; and 14 of 50 *MeMAPKKKs* displayed relatively higher transcripts in leaves, compared with that in stems or storage roots. For W14 subspecies, 12 of 50 *MeMAPKKKs* showed higher transcripts in storage roots, relative to that in stems and leaves; and 23 of 50 *MeMAPKKKs* displayed relatively higher transcripts in leaves, compared with that in stems or storage roots. Besides, some *MAPKKK* genes had differential expression patterns between the two accessions, such as *MeMAPKKK-1*, *-11*, *-16*, *-19*, *-20*, *-29*, *-48*, *-52*, *-58* showing high transcript abundance (value >5) in W14 stems, whereas exhibiting low expression (value <5) in Arg7 stems, suggesting their differential roles in different organs of the two cassava accessions. Taken together, the tissue expression patterns of *MAPKKK* genes in different genotypes may provide valuable information for further study of organ development and function.

### Expression profiles of *MeMAPKKK* genes after drought treatment in three cassava accessions

**Figure 5:**
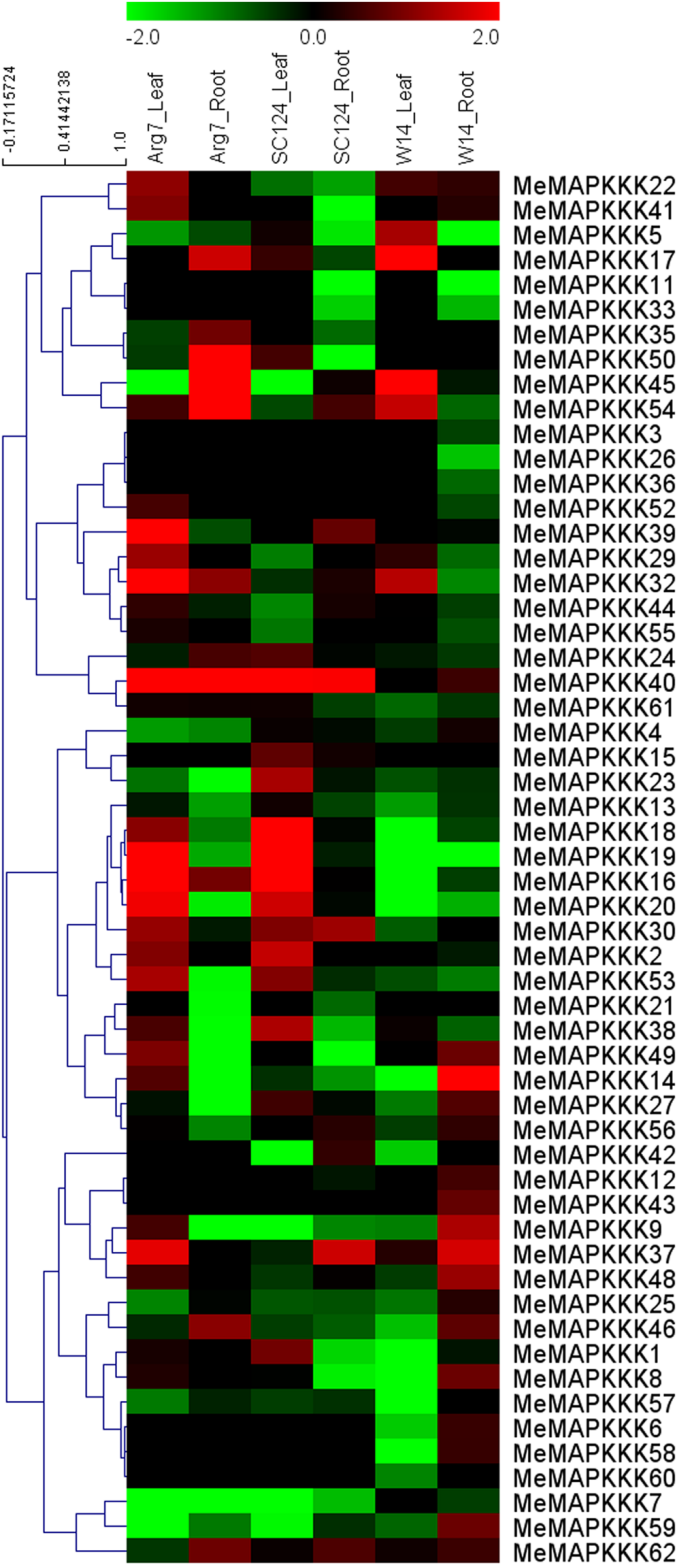
Expression profiles of cassava *MAPKKKs* in response to drought stress in leaves and roots of Arg7, SC124, and W14. Log2 based fold change was used to create the heat map. Fold changes in gene expression are shown in color as the scale.

To seek insights into the clues of *MeMAPKKKs* in cassava response to drought, three accessions of cassava seedlings were exposed to drought conditions for 12 days, and then the leaves and roots were sampled to perform RNA-seq analyses (Fig. 5; Table S3). From the results, 56 *MeMAPKKKs*, except for *MeMAPKKK10*, *-28*, *-31*, *-34*, *-47*, *-51*, showed the corresponding transcriptomic data.

In the Arg7 variety, 7/56 and 14/56 *MeMAPKKKs* were induced (log2-based values >1) after drought stress in roots and leaves, respectively, with two genes (*MeMAPKKK32* and *MeMAPKKK40*) showing upregulation in both roots and leaves. In the SC124 variety, 3/56 and 9/56 of *MeMAPKKK*s expression increased after drought stress in roots and leaves, respectively, with *MeMAPKKK40* showing upregulation in both roots and leaves. In W14 subspecies, 4/56 and 5/56 of *MeMAPKKK*s genes showed induction after drought stress in roots and leaves, respectively. These results suggested that the number of *MeMAPKKKs* upregulated by drought was greater in Arg7 and SC124 than those in W14.

Besides, some *MeMAPKKKs* showed similar expression profiles in SC124 and Arg7, which is different from W14. Seven genes (*MeMAPKKK2*, -*16*, -*18*, -*19*, -*20*, -*40*, -53) were upregulated in leaves of Arg7 and SC124, whereas were downregulated or no response in leaves of W14 after drought treatment. We also found that two genes (*MeMAPKKK9*, -14) showed upregulation in roots of W14, but repression in roots of Arg7 and SC124. Thus, the *MAPKKK*s mediated mechanisms of drought response may be differentiated between wild subspecies and cultivated varieties.

### Expression patterns of *MeMAPKKK* genes under various stress and related signalling treatments

**Figure 6:**
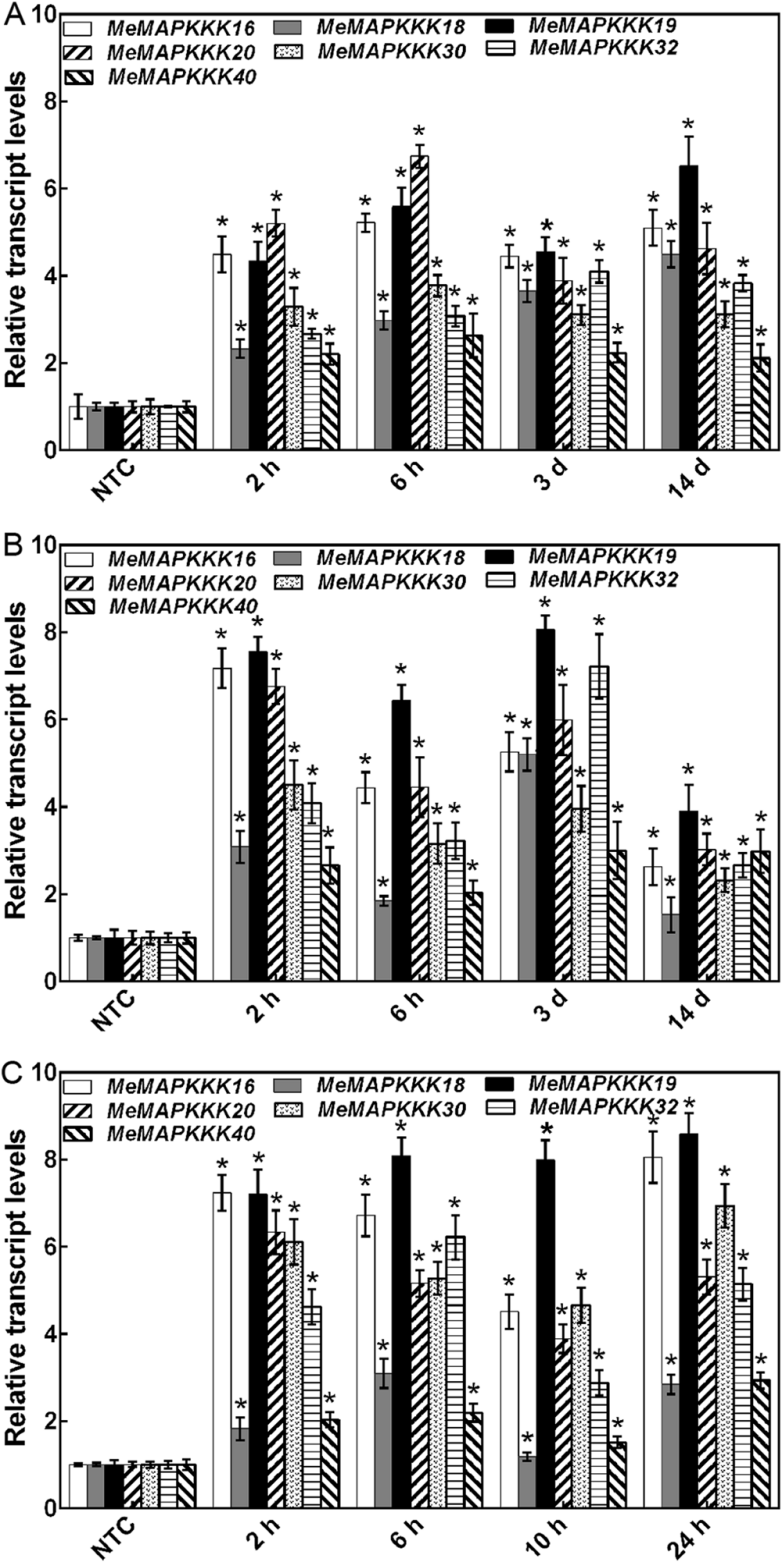
Expression patterns of selected *MeMAPKKKs* after osmotic (**A**), salt (**B**), and ABA (**C**) treatments in cassava. The mean fold changes of each gene between treated and control samples at each time point were used to calculate its relative expression levels. NTC indicates no treatment controls (mean value = 1). Data are means ± SD of n = 3 biological replicates.

**Figure 7:**
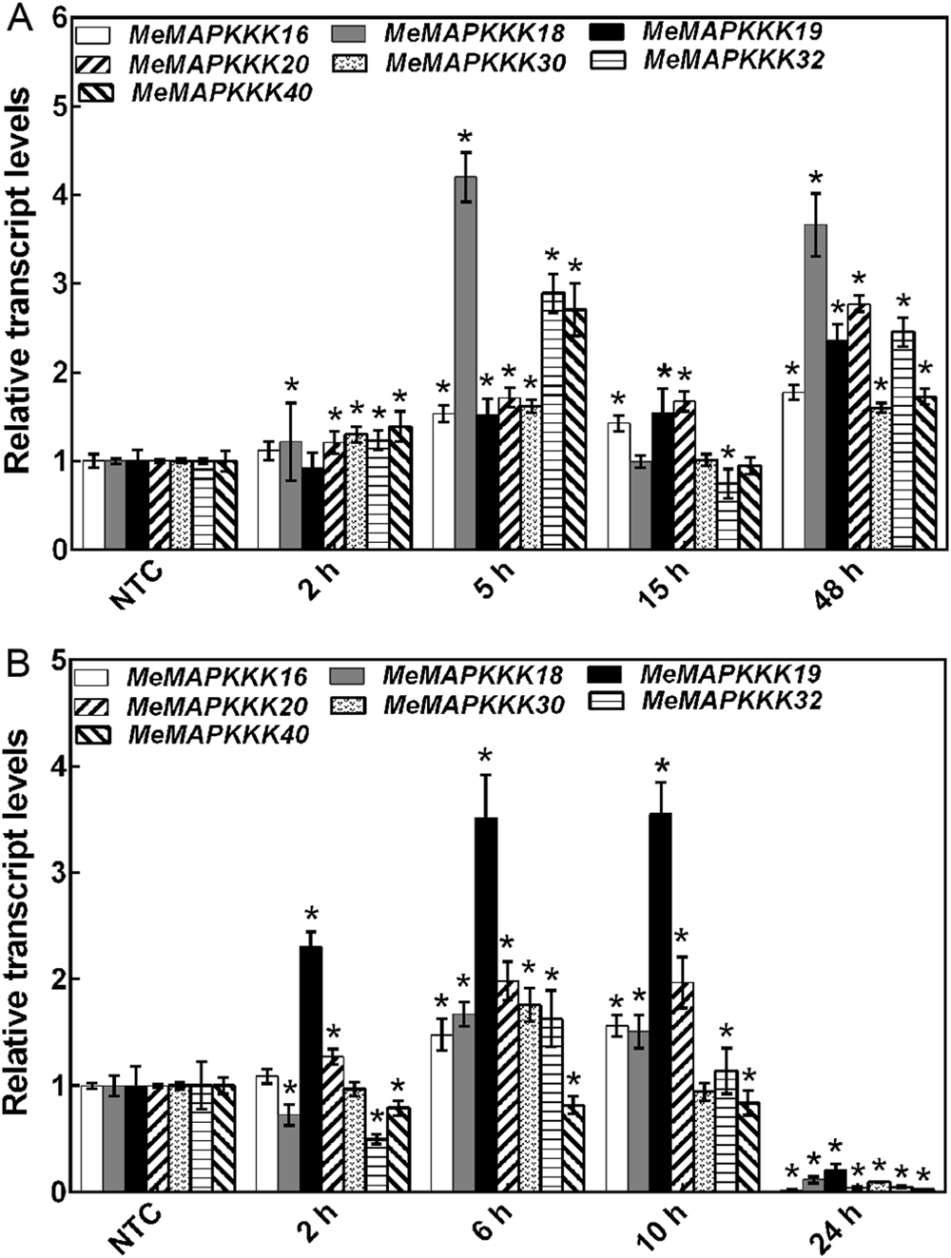
Expression patterns of selected *MeMAPKKKs* after cold (**A**) and H_2_O_2_ (**B**) treatments in cassava. The mean fold changes of each gene between treated and control samples at each time point were used to calculate its relative expression levels. NTC indicates no treatment controls (mean value = 1). Data are means ± SD of n = 3 biological replicates.

**Figure 8:**
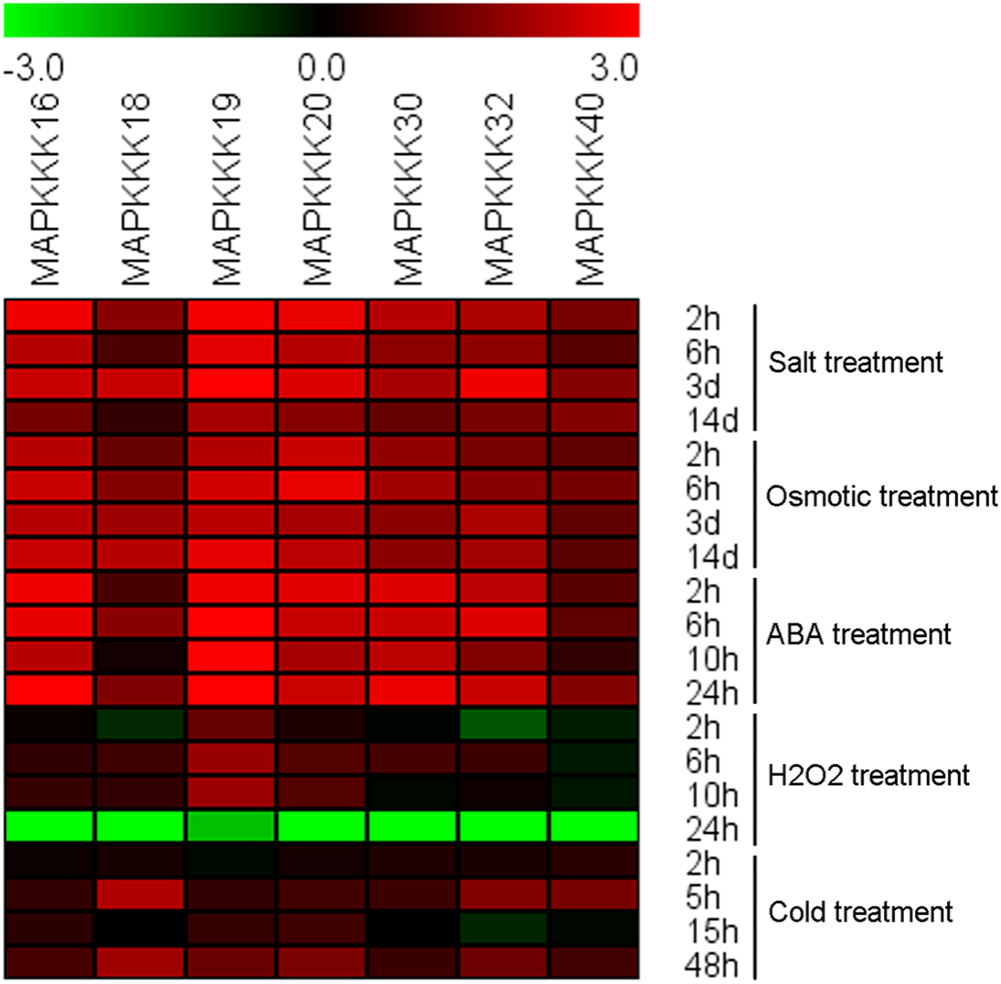
Expression of cassava selected *MeMAPKKKs* in response to various stimuli. The heatmap was constructed using Log2 based fold change from three biological replicates of qRT-PCR data. The scale represents the relative signal intensity.

According to the RNA-seq data, 7 *MeMAPKKK* genes (*MeMAPKKK*-16, -18, -19, -20, -30, -32 and -40) upregulated by drought were chosen to investigate their expression after salt, osmotic, cold, ABA and H_2_O_2_ treatments (Figs 6–8; Table S4). In response to osmotic, salt and ABA treatments, all the tested *MeMAPKKK*s were significantly up-regulated during the whole treated time points (Figs 6 and 8). Under cold stress condition, *MeMAPKKK20* transcript was significantly induced during all the tested time points, and the other 6 *MeMAPKKK*s showed upregulation at several time points (Figs 7A and 8). After H_2_O_2_ treatment, *MeMAPKKK40* displayed significant downregulation at all time points, whereas the other 6 genes were slightly induced at several time points (Figs 7B and 8). Together, these results indicated that most of the *MeMAPKKKs* could be significantly upregulated by osmotic, salt, and ABA treatments, whereas slightly affected by cold and H_2_O_2_ treatments (Fig. 8; Table S4), suggesting that *MeMAPKKKs* may participate in multiple signalling transduction pathways in cassava.

## Discussion

Due to the central role of MAPK cascade in plant development and stress response and the characteristic of drought resistance of cassava, it is necessary to investigate the possible role of MAPKKKs in cassava. So far, there is no information for the MAPKKK family in cassava. In the present study, 62 MeMAPKKK genes were identified from cassava. Previously, genome-wide analyses have identified many MAPKKKs in various plant species, including 80 members in *Arabidopsis* ^23^, 75 in *Oryza sativa* ^24^, 71 in *Zea mays* ^25^, 89 in *Lycopersicon esculentum* ^26^, 59 in *Cucumis sativus* ^27^, 150 in *Glycine max* ^28^, 45 in *Vitis vinifera* ^31^, 73 in *Medicago truncatula* ^29^, and 77 in *Musa acuminate* ^30^. This indicated that MAPKKKs in cassava had expanded compared to that in *Cucumis sativus* and *Vitis vinifera*, while had shrunk compared to that in *Arabidopsis*, *Oryza sativa*, *Zea mays*, *Lycopersicon esculentum*, *Glycine max*, *Medicago truncatula*, and *Musa acuminate*. Cassava MAPKKKs were grouped into three subfamilies together with Arabidopsis MAPKKKs based on the evolutionary analysis (Fig. 1). This is in line with the previous evolutionary analysis of MeMAPKKKs from Arabidopsis, rice, tomato, and cucumber^3,24,26,27^. To better understand the evolution of MAPKKK family, 13 members from the ancestral *Chlamydomonas reinhardtii* were acquired using Phytozome V10.0 database. The evolutionary investigation indicated that most *Chlamydomonas reinhardtii* MAPKKKs (9 members) were clustered into Raf subfamily, implying that Raf subfamily may represent the original members of MAPKKK family (Fig. 1). We also observed that the Raf subfamily showed significantly more introns than the ZIK and MEKK subfamilies in cassava (Fig. 2). Previous report suggested that the rate of intron loss is faster than the rate of intron gain after segmental duplication^37^. This allows us to infer that Raf subfamily may contain the original genes, from which those in other subfamilies were derived. Intron-exon organization analyses showed that the number of intron in *MeMAPKKKs* was highly variable, varying from 0 to 16 (Fig. 2). The large-scale variation in structures of *MeMAPKKKs* implied that the cassava genome has significantly variable during its long evolutionary history. This phenomenon is also observed in maize (from 1 to 17) and grapevine (from 4 to 16)^25,31^. Conserved motif and gene structure analyses suggested that MeMAPKKKs in the same subfamily showed similar conserved motifs and exon-intron structures, further supporting the classification of MeMAPKKKs (Figs 2 and 3). Moreover, all the identified MeMAPKKKs had the typical protein kinase domain, which was also confirmed in other plants, including Arabidopsis, rice, cucumber, and tomato^3,24,26,27^.

Although cassava has strong resistance to drought stress, the underlying mechanism is less known. Previous studies demonstrated the positive role of MAPKKKs in plants resistant to abiotic stress, including drought, salt, and cold^11,19,21,22^. In the present study, we found that many MeMAPKKK genes showed induction after drought treatment in three cassava accessions, indicating their possible role in the regulation of cassava resistance to drought stress. By comparing the expression profiles of MeMAPKKK genes in different cassava accessions, we found that the number of *MeMAPKKKs* upregulated by drought was greater in leaves of Arg7 (14/56) and SC124 (9/56) than those in W14 (5/56) (Fig. 5). W14 is a drought resistant genotype, which is more resistant than Arg7 and SC124^33–36^. Some studies have revealed the positive role of MAPKKKs in drought response in Arabidopsis, rice, and maize by biochemical and genetic approaches^19,22,38,39^. Based on the above results, we speculated that the cultivated varieties (SC124 and Arg7) need to activate more MAPKKK genes in response to drought stress, thus well-fitting the drought environment, compared to wild subspecies W14. Notably, some *MeMAPKKKs* showed different expression profiles between SC124/Arg7 and W14, indicating the MAPKKK mediated drought resistance mechanism may be differentiated between wild subspecies and cultivated varieties.

As the core regulation components of MAPK cascade, MAPKKK widely participates in plants response to abiotic stress^11,19,21,22^. Based on the transcriptomic data, some MeMAPKKK genes could respond to drought stress. Therefore, it is essential to investigate the possible role of MeMAPKKK genes under various abiotic stress and stress-related signal molecule treatments. Interestingly, all the tested 7 *MeMAPKKK* genes (*MeMAPKKK*-16, -18, -19, -20, -30, -32 and -40) were significantly upregulated after osmotic, salt and ABA treatments during the whole treated time points, indicating that these genes may be the cross points of multiple stress signaling (Figs 6 and 8). In banana, 13.2% and 2.6% *MAPKKKs* were significantly induced by osmotic and salt stress in BX variety, and 15.8% and 5.3% *MAPKKKs* were upregulated under each stress in FJ variety^30^. In grapevine, most of the *MAPKKK* genes showed induction after 8 d and 12 d drought treatment^31^. In tomato, most of the *MAPKKK* genes were upregulated by drought, salt, cold, and heat stress, among which *SlMAPKKK51*, *SlMAPKKK53*, and *SlMAPKKK55* were upregulated by more than 100-fold after heat or drought treatment^26^. Further biochemical and genetic studies revealed that some *MAPKKK* members, including *GhRaf19*, *AtMAPKKK18*, *AtRaf43*, and *DSM1* (a rice *MAPKKK*) were involved in plants resistance to abiotic stress^21,38–40^. These results suggested that *MAPKKK* family genes could widely participate in abiotic stress response. Moreover, MAPK cascade is considered to be crucial components of the ABA signaling transduction network^39–42^. Recently, the MAP3K17/18-MKK3-MPK1/2/7/14 module was found to be under the control of the ABA core signalling pathway at transcriptional and post-translational levels^42^. Due to the crucial role of ABA signalling in plants response to abiotic stress, these *MeMAPKKK* genes may be involved in ABA mediated abiotic stress response. In contrast, most of the tested *MeMAPKKK* genes were slightly affected by cold treatments (Fig. 7A), which could be explained by the warm habitat and cold-sensitive characteristic of cassava^43,44^.

In conclusion, this study identified 62 MAPKKKs from cassava and investigated their classification, protein motif, and gene structure. Organ expression analysis indicated the different expression profiles of *MeMAPKKK* genes in two different accessions. Transcriptomic analysis revealed the possible role of *MeMAPKKK* genes against drought stress in different cassava genotypes. Finally, several *MeMAPKKK* genes were identified as important candidates for improving crop resistances to multiple stresses. Together, these results will advance the understanding of *MeMAPKKK*-mediated abiotic stress response and provide candidate *MeMAPKKK* genes used for genetic improvement of crop resistance to abiotic stress.

## Methods

### Plant materials and treatments

The characteristic of W14, SC124 and Arg7 was described in previous studies^45,46^. Segments of cassava stems were taken from mother plants, and cultured in pots in a growth room with a 16 h/35 °C day and 8 h/20 °C night regime, and a relative humidity of 70%. Then, 90-day-old stems, 90-day-old leaves, and 150-day-old tuberous roots were acquired from W14 and Arg7 under normal conditions to study the expression levels of *MeMAPKKK*s in distinct organs. To detect the transcriptional changes of *MeMAPKKK*s in response to drought, roots and leaves were collected from W14, SC124, and Arg7, respectively, under drought conditions for 12 d. For osmotic, cold, salt, ABA and H_2_O_2_ treatments, two-month-old seedlings of Arg7 were suffered from 200 mM mannitol for 14 d, 300 mM NaCl for 14 d, 100 µM abscisic acid (ABA) for 24 h, 10% H_2_O_2_ for 24 h and low temperature (4 °C) for 48 h, respectively.

### Identification and evolutionary analyses

Protein sequences of AtMAPKKKs and OsMAPKKKs were downloaded from RGAP and UniPort databases, respectively^47,48^. The candidate MeMAPKKKs were firstly acquired with local hidden Markov Model-based search and BLAST search from cassava genome database using Arabidopsis and rice MAPKKKs as queries^49,50^. Subsequently, the candidate MeMAPKKKs were further checked with PFAM and CDD dataases^51,52^. Then, the protein sequences of 62 MeMAPKKKs and 80 AtMAPKKKs, and 13 MAPKKKs from *Chlamydomonas reinhardtii* were used to construct a neighbor-joining (NJ) phylogenetic tree using Clustal X2.0 and MEGA 5.0 software with 1000 bootstrap replicates^53,54^.

### Protein properties and sequence analyses

ExPASy proteomics server was used to predict the molecular weight (MW) and isoelectric points (pI) of cassava MAPKKK family proteins^55^. MEME program was used to identify the conserved proteins motifs^56^. Furthermore, all identified motifs were annotated according to InterProScan^57^. The gene structures were identified using GSDS database^58^.

### Transcriptomic analysis

Total RNA of each sample was extracted with plant RNA extraction kit (TIANGEN, China) and used for cDNA library construction. The sequencing was performed with an Illumina GAII following manufacturer’s instructions. Adapter sequences were removed with FASTX-toolkit. Clean reads were generated by removing low quality sequences using FastQC. Tophat v.2.0.10 was used to map the clean reads to the cassava genome. Using cufflinks, the transcriptome data was assembled^59^. Genes were scored as not expressed if the corresponding RNA-seq reads could not align to the genome. Gene expression levels were calculated as Reads Per Kilobase of exon model per Million mapped reads (FPKM). DEGseq was used to identify differentially expressed genes in response to drought^60^. The transcriptiomic data was submitted to NCBI and the accession number was listed in Supplementary Table S5.

### Quantitative RT-PCR analysis

The leaf samples of Arg7 in response to salt, osmotic, cold, ABA and H_2_O_2_ were collected to isolate total RNA. Then, the expression of *MeMAPKKKs* was detected by qRT-PCR analysis using TransStart Green qPCR SuperMix (TRANS, Beijing, Chian) chemistry on a Stratagene Mx3000 P (Stratagene, CA, USA) instrument. Agarose gel electrophoresis, melting curve, and sequencing analyses were performed to confirm the specificities of primer pairs. The primers of target genes were listed in Supplementary Tables S6. For each target gene, expression data were normalized with expression levels of β-tubulin gene (TUB) and elongation factors 1α gene (EF1) and calculated by the formula 2^−ΔΔCt^ method^61,62^.

## Electronic supplementary material

Dataset 1
